# The Associations of Single Nucleotide Polymorphisms with Risk and Symptoms of Irritable Bowel Syndrome

**DOI:** 10.3390/jpm12020142

**Published:** 2022-01-21

**Authors:** Tingting Zhao, Yiming Zhang, Joochul Lee, Angela R. Starkweather, Erin E. Young, Xiaomei Cong

**Affiliations:** 1 School of Nursing, University of Connecticut, Storrs, CT 06269, USA; tingting.zhao@uconn.edu (T.Z.); angela.starkweather@uconn.edu (A.R.S.); 2 Department of Statistics, University of Connecticut, Storrs, CT 06269, USA; yiming.3.zhang@uconn.edu; 3 Department of Biostatistics and Epidemiology, University of Pennsylvania, Philadelphia, PA 19104, USA; joochul.lee@pennmedicine.upenn.edu; 4 Department of Anesthesiology, University of Kansas Medical Center, Kansas City, KS 66160, USA; eyoung6@kumc.edu

**Keywords:** irritable bowel syndrome, single nucleotide polymorphisms, sleep disturbance, fatigue, pain interference

## Abstract

Although several risk single nucleotide polymorphisms (SNPs) have been found to play an important role in etiology of irritable bowel syndrome (IBS), the findings are inconsistent. A descriptive correlational design was used to analyze the baseline data of a randomized controlled trial including participants with IBS and healthy controls (HC). Pain severity and interference, anxiety, sleep, and fatigue were measured using the Brief Pain Inventory (BPI) and patient-reported outcomes measurement information system (PROMIS). Fisher’s exact test and multivariate linear regression were used to investigate the associations between IBS risk alleles and IBS symptoms. Participants were predominantly female, white, and had an average age of 21.13 ± 2.42 years. Polymorphisms within *TNFSF15* (rs4263839), *SLC6A4* 5-HTTLPR, *HTR3A* (rs1062613), and *OXTR* (rs2254298) were associated with IBS risk, and *TNFSF15* (rs4263839), *COMT* (rs6269), *SLC6A4* 5-HTTLPR polymorphisms were associated with pain severity. *TNFSF15* (rs4263839) and *COMT* (rs4680; rs4633) genotypes were associated with sleep disturbance, and the *ADRA1D* SNP rs1556832 was associated with fatigue in both IBS and HC groups. Genotypic differences were associated with IBS risk and symptoms including abdominal pain, sleep disturbance, and fatigue. Further investigation is warranted to reveal the mechanisms by which these genetic variations influence the dynamic nature of IBS symptoms over time.

## 1. Introduction

Irritable bowel syndrome (IBS) is the most common functional gastrointestinal disorder, with a global prevalence of 7–21% [1,2]. Gastrointestinal (GI) and psychological symptoms such as abdominal pain, stress, anxiety, fatigue, and sleep disturbances impact the quality of life (QOL) of young adults with IBS [3,4,5,6,7,8,9]. Although there have been advances in treatment, 62% of patients with IBS under the care of a gastroenterologist remain with or have worsening symptoms [10]. The etiology of IBS is still largely unknown, and its treatment needs to be more precise and personalized under the guide of biomarkers exploration. More than 65 single nucleotide polymorphisms (SNPs) have been found to be associated with IBS symptom severity [11], including anxiety, fatigue, sleep disturbance, and pain [12,13,14], although some of the findings have failed to withstand replication [15]. Exploring the associations between genotypic variations and IBS risk and/or symptom burden targeting the underlying mechanisms at work is critical to developing more precise and effective treatment modalities.

The tumor necrosis factor superfamily member 15 (*TNFSF15*), encoding the protein of the same name, is associated with inflammatory pain in patients with IBS [16,17,18]. *TNFSF15* is expressed in epithelial cells, including those that line the intestinal walls [19]. The genotype of SNP *TNFSF15* rs4263839 (located at 9q32) correlated with *TNFSF15* mRNA expression was associated with an increased risk of IBS [15,16,20]. Although these genetic polymorphisms alone are not sufficient for the IBS diagnosis, these variations suggest a role of *TNFSF15* in the pathology of IBS and the severity of IBS symptoms, e.g., sleep disturbance [21]. TNF has been found to be associated with sleep regulation in preclinical and clinical studies [13], even though the mechanism of the association between sleep disturbance and the IBS is still underdeveloped. Prior work also indicates that TNF levels are decreased during sleep deprivation [22], but there is still a lack of studies to confirm this association in IBS patients.

Dysregulation of catecholaminergic signaling has been implicated in the etiology of IBS, as supported by reports that SNPs within two catecholaminergic signaling pathway genes, Catechol-O-methyltransferase [23] and alpha adrenergic-1D (*ADRA1D*), are associated with IBS symptom severity [24]. COMT, the protein encoded by the *COMT* gene, is involved in the breakdown of catecholamines (dopamine, noradrenaline, and adrenaline). *COMT* SNP genotype is highly predictive of subsequent COMT enzymatic activity and, therefore, catecholamine levels in the brain and circulation [25]. COMT plays an essential role in the degradation of norepinephrine and dopamine, associated with pain threshold and pain perception [25,26]. Presence of the *COMT* rs4680 (val158met) major allele has been reported to be associated with increased chronic pain and anxiety in IBS patients [27]. In addition, Val allele carriers exhibit improved IBS symptoms through cognitively focused intervention compared to patients homozygous for the Met allele, which has been shown to decrease COMT activity [25]. Further supporting the role of catecholamine signaling in determining IBS symptom severity, SNP *ADRA1D* or Alpha-1A adrenergic receptor (*ADRA1A*) [28] rs1556832 has been proposed as having interactions with *COMT* and alpha-adrenergic-β2 (*ADRAβ2*) to worsen the severity of IBS gastrointestinal symptoms [24].

Polymorphisms in the 5-serotonin-transporter-linked polymorphic region (5HTTLPR) of the serotonin reuptake transporter (SERT) gene (*SLC6A4*) have been found to be associated with IBS symptom burdens [29]. Three different 5-HTTLPR alleles-short (s) allele (484bp), long (l) allele (528bp), and extra-long (xl) allele (572bp) developed during *SLC6A4* 5-HTTLPR’s deletion and insertion play an important role in promoter activity regulation [30]. 5-HTTLPR polymorphism in *SLC6A4* gene promoter may influence SERT expression and then regulate serotonin function by adjusting intracellular levels of serotonin [31]. It has also been found that the interaction of polymorphisms of *COMT* rs4680 and s allele at *5-HTTLPR* resulted in a gray matter volume change, which was associated with increased depression risk [32,33]. The influence of serotoninergic signaling in the etiology of IBS has been further shown from studies indicating an association between hydroxy tryptamine receptor 3A encoding the 5-HT3 receptor (*HTR3A*) rs1062613 SNP genotype and increased risk of IBS [34]. The polymorphism of *HTR3A* rs1062613 is located within a cis-regulatory region, which may impact *HTR3A* subunit expression [34]. Two polymorphic variations of oxytocin receptor (*OXTR*) rs2254298 and rs53576 located at intron 3 were associated with depression [35,36]. The genetic variant of *OXTR* has been recently reported in IBS patients compared with people in the healthy control group [37], but further studies are needed to explore the role of *OXTR* in the pathology of IBS. The gene of *Opioid Receptor Mu 1* (*OPRM1*) encodes the opioid receptor, which plays an important role in GI disfunction and disorder [38]. The polymorphic variation (rs1799971) of *OPRM1* located at exon 1 impacts the potency of the opioid receptor [39], although the role of this SNP in IBS severity and symptoms is still unknown.

To date, the etiology of IBS is still unclear but is thought to arise from a combination of alterations involving brain-gut interaction and environmental factors. Further, effective treatment of IBS is challenging, although therapeutic effects of selective serotonin reuptake inhibitors (SSRIs), TNF inhibitors, anticholinergics, and dietary and lifestyle modifications in patients with IBS have been reported [40,41]. In our study, we want to understand the driving factors behind the IBS risk and the severity of IBS symptoms. Therefore, we selected candidate polymorphisms of *TNFSF15* rs4263839, *OXTR* rs2254298 and rs53576, *OPRM1* rs1799971, *HTR3A* rs1062613, *COMT* rs4680, rs4818, rs6269, rs4633, and *ADRAID* rs1556832 as susceptibility loci either related to GI disorders or symptoms such as sleep deprivation, pain, anxiety, and depression [20,21,34,35,36,37,38,39]. We hypothesize that there exists an association between polymorphisms in our candidate genes and IBS symptoms. Our study aims to identify IBS risk alleles and explore the associations between candidate alleles and IBS-related symptoms. Risk alleles can be considered biological markers of IBS and symptom severity, and future studies can be conducted to develop the putative therapeutic targets.

## 2. Methods

### 2.1. Settings and Participants

A descriptive correlational design was used, and the baseline data of a randomized controlled trial were analyzed (RCT registration: ClinicalRrial.gov-Protocol ID: H16-152), including young adults with IBS [42]. This study was approved by the Institutional Review Board of University of Connecticut (H16-152, 20 April 2017). The study participants were recruited from the community, two large public university campuses, and two gastrointestinal (GI) clinics from a region in the northeastern U.S.

In the IBS group, 80 subjects (age 18–29) were recruited, and the sample size was determined in our clinical trial [42]. Inclusion criteria for the IBS participants included: (1) diagnosis of IBS by a healthcare provider and symptomatic at the time of enrollment; (2) able to speak and read English; (3) access to a computer, smartphone, or tablet with an internet connection. Exclusion criteria included: (1) other gastrointestinal disorders or other forms of chronic visceral pain; (2) infectious diseases (e.g., hepatitis, HIV, MRSA); (3) celiac disease or inflammatory bowel disease; (4) diabetes mellitus; (5) mental health diagnosis or under care for mental health problems; (6) pregnancy or postpartum within 3 months; (7) using medication to control symptoms (opioids, iron supplements, prebiotics or probiotics, antibiotics, substance abuse); and (8) any ongoing injury or disease that may influence symptoms of IBS. In the healthy control group, 21 healthy participants were recruited using a convenience sampling method following the inclusion criteria: men and women aged 18–29, without any acute or chronic disease or any current pain condition.

### 2.2. Measurement and Data Collection

In both IBS and the healthy control (HC) groups, demographic characteristics, pain severity, sleep condition, and fatigue were measured. Buccal swabs were collected from all subjects and eleven candidate polymorphisms of *TNFSF15* rs4263839, *ADRA1D* rs1556832, *COMT* (rs4680, rs4818, rs6269, and rs4633), *SLC6A4* 5-HTTLPR, *HTR3A* rs1062613, *OPRM1* rs1799971, and *OXTR* (rs53576 and rs2254298) were assessed.

Demographic data: The subjects’ age, sex, race, ethnicity, educational level, caregiver primary type, employment status, and marital status were collected in both IBS and healthy control groups using a demographic questionnaire.

Brief pain inventory (BPI): The BPI scale (each item scoring from 0 = no pain to 10 = worst pain) was used to assess pain severity (four items with total scores from 0 to 40) and pain interference (seven items with total scores from 0 to 70) [43]. Four items to assess pain severity include worst pain in the last 24 h, least pain in the last 24 h, average pain, and pain right now. Mean BPI pain severity was calculated by adding four pain scores together and divided by four [44]. Seven items to assess pain interference include general activity, mood, walking ability, normal walk, relations with other people, sleep, and the enjoyment of life [45].

Patient-reported outcomes measurement information system (PROMIS): the PROMIS system was initially developed by National Institutional of Health (NIH) in 2004 to assess patients’ physical, mental, and sociological health functions [46]. PROMIS was widely applied in different populations with chronic diseases, including patients with IBS [47]. The PROMIS Item Bank Adult Short Form 6a V1.0 was used in our study to measure five domains including fatigue, anxiety, depression, applied cognition, and sleep disturbance in both IBS participants and the healthy control group. Each domain has 6 items with total raw scores from 6 to 30 or T-scores from 39.1 to 82.7 [48].

Pain-susceptibility SNPs genotyping: Genotyping of selected pain-sensitive SNPs was conducted by using buccal swab samples. Participants were instructed to rinse their mouths twice with water and then collect the buccal cells by firmly brushing the inside of the cheek. Buccal samples were kept in the −80 °C freezer of our Biobehavioral Laboratory for further analysis. Genomic DNA was extracted from buccal samples using Gentra Puregene Buccal Cell Kit following the protocol (#158845). Polymorphisms of *TNFSF15* rs4263839, *ADRA1D* rs1556832, *COMT* (rs4680, rs4818, rs6269, rs4633), 5-HTTLPR in *SLC6A4*, *HTR3A* rs1062613, *OPRM1* rs1799971, and *OXTR* (rs53576, rs2254298) were assessed. Taqman SNP genotyping assays (VIC/FAM) and allelic discrimination analysis was conducted using an Applied Biosystems StepOnePlus^TM^ PCR machine, and StepOne^TM^ and StepOnePlus^TM^ software v2.0 (Thermo Fisher Scientific, Waltham, MA, USA) [42].

### 2.3. Data Analysis

We performed data management and statistical analysis using the R software (version 4.0.2, R Foundation, Vienna, Austria). Demographic characteristics and the measurements for pain and psychological symptoms were summarized by descriptive statistics for both IBS and HC groups. To examine proportions of demographics according to the different groups, we performed a chi-squared test for sex and Fisher’s exact test for race, ethnicity, education, caregiver type, employment status, and marital status. A Wilcoxon rank-sum test was conducted to check the differences of age and the pain measurements between two groups. The chi-square test of the Hardy-Weinberg Equilibrium (HWE) was conducted for each SNP in the IBS and the HC groups, respectively. We investigated the association between IBS risk and SNPs by checking the difference of distribution of genotypes for each SNP between the IBS and the HC groups. Due to the small sample size of certain genotypes, robust statistical methods were considered for statistical inference and calculating odds ratios (OR) of IBS risk. For each SNP, we performed Fisher’s exact test to check the association between IBS risk and genotype groups. Multiplicity adjustment using Holm-Bonferroni correction was applied for the SNPs on the same gene. We had no prior hypotheses as to which genetic model would be the most appropriate, so the median-unbiased (mid-p) confidence intervals (CI) of ORs were computed for all 5 gene models to investigate a potential relationship between SNPs genotypes and IBS risk. The five gene models are allele model, dominant model, recessive model, homozygous model, and heterozygous model.

Linear regression analysis was performed to investigate the association between pain, psychological symptoms and SNPs. Due to the small sample size, we assumed the effect of the number of risk alleles in each SNP was linear to the outcome measurements and used the number of risk alleles as a predictor to improve the power of hypothesis testing in linear regression [49]. In addition, we adjusted sex, race, ethnicity, and type of caregiver in the models to control the potential confounding effects. For the linear regression models for IBS pain measurements, only the participants in the IBS group were included, since participants in the HC group did not have pain. For the analyses of psychological symptoms, both IBS and HC groups were used in the linear regression models, and the group was considered an additional adjusting covariate.

## 3. Results

### 3.1. Demographic Characteristics

Table 1 summarizes the demographic characteristics. The majority of the participants were female (71.29%), white (72.28%), non-Hispanic (83.17%), unmarried (97.03%), and college students (74.26%). There were no significant differences in demographic characteristics between the IBS (*n* = 80) and HC (*n* = 21) groups.

### 3.2. IBS Related Pain and Psychological Symptom Measurements

The descriptive analysis of pain and psychological symptom measurements are presented in Table 2. Not surprisingly, the IBS group reported significantly higher BPI average pain severity (*p* = 1.6 × 10^−10^) and BPI interference (*p* = 5.1 × 10^−9^) than the HC group. The IBS participants also reported higher anxiety (*p* = 0.005), fatigue (*p* = 0.003), and sleep disturbance (*p* = 0.051) scores compared to those in the HC group. There were no significant differences in cognition concern and depression scores between the two groups.

### 3.3. Hardy-Weinberg Equilibrium Test

The HWE analysis using chi-square tests are presented in Supplementary Table S1. For all SNPs assessed, we found the control group to be in HWE except for HTR3A rs1062613, while for our phenotype of interest (IBS), two SNPs, HTR3A rs1062613 and OPRM1 rs1799971, were out of HWE. This is not surprising, given that we selected these SNPs for their potential association with IBS which would be supported by an over-representation of risk alleles in that group. These findings likely reflected the influence of individual SNPs on the outcomes of interest and the impact of small deviations from expected population level allele frequencies in our small sample size.

### 3.4. Associations between IBS Risk and SNP Genotypes

**Figure 1:**
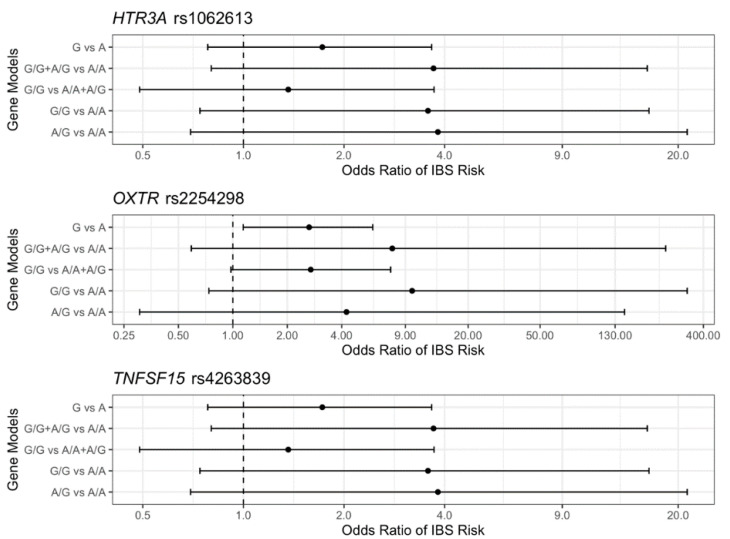
Mid-p estimates and 95% confidence intervals of odds ratio (ORs) of irritable bowel syndrome (IBS) risk for the gene models in (single nucleotide polymorphisms) SNPs of hydroxy tryptamine receptor 3A encoding the 5-HT3 receptor (*HTR3A)* rs1062613, oxytocin receptor (*OXTR)* rs2254298, and tumor necrosis factor superfamily member 15 (*TNFSF15)* rs4263839.

The frequency and proportion of genotypes of the 11 SNPs in IBS and HC groups, as well as the results of Fisher’s exact test, are presented in Supplementary Table S1. We observed significant differences of genotype distribution between IBS and HC groups in *SLC6A4* 5-HTTLPR (*p* < 0.001) and *HTR3A* rs1062613 *(p* = 0.014). For *SLC6A4* 5-HTTLPR, most of the participants in the HC group carried the s allele (95.24%), while the majority of participants in IBS group carried l or xl alleles (76.25%). To identify SNPs associated with IBS risk, Figure 1 visualizes the ORs of higher IBS risk in different gene models and their 95% CI. We observed higher IBS risk in the dominant model (C/C + C/T vs. T/T, OR = 3.64, 95% CI = (1.19, 11.03)) of *HTR3A* rs1062613 and the allele model (G vs. A OR = 2.64, 95% CI = (1.14, 5.94)) of *OXTR* rs2254298. We also observed trends of increased IBS risk in the gene models with C allele in *HTR3A* rs1062613, with G allele in *OXTR* rs2254298, and with G allele in *TNFSF15* rs4263839.

### 3.5. Associations between IBS Related Pain Severity and SNP Genotypes

**Figure 2:**
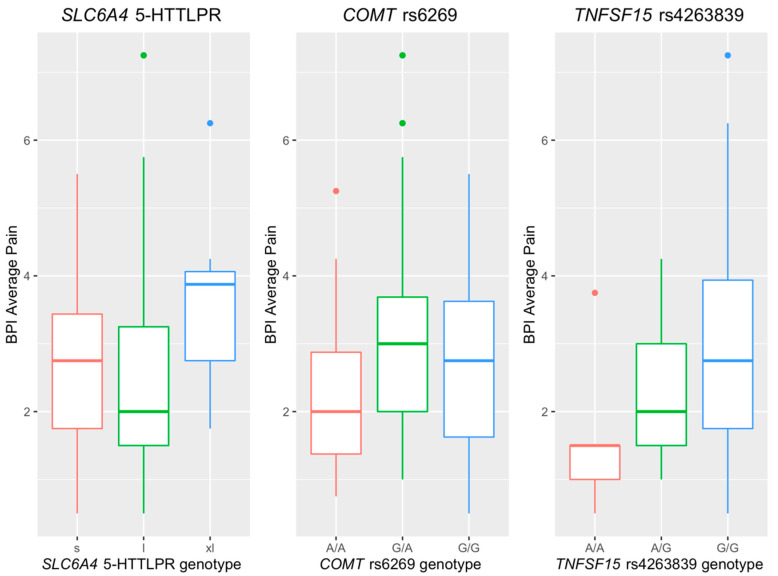
Boxplots for BPI average pain in the polymorphisms of *SLC6A4* 5-HTTLPR, *COMT* rs6269, and *TNFSF* rs4263839.

Among the 11 polymorphisms, 5-HTTLPR in *SLC6A4*, *COMT* rs6269, and *TNFSF15* rs4263839 were significantly associated with the BPI average pain severity. Figure 2 visualizes the marginal difference in BPI average pain severity across the different genotypes of the three SNPs. For 5-HTTLPR in *SLC6A4*, we observed a higher BPI average pain severity in the xl allele carriers compared to the IBS participants carrying s and l alleles. For *COMT* rs6269, higher BPI average pain severity was reported by the G allele carriers compared to the IBS participants carrying A/A homozygous. In addition, a trend of increased BPI average pain severity was observed in the participants carrying one more G allele of *TNFSF15* rs4263839. Table 3 presents the results of linear regression analysis of BPI average pain severity with respect to the three SNPs mentioned above. Compared to the participants carrying l and s alleles of 5-HTTLPR in *SLC6A4*, the xl carriers reported 1.24 (*p* = 0.013, 95% CI = (0.27, 2.22)) higher BPI average pain severity. Additionally, the BPI average pain severity increased by 0.41 (*p* = 0.045, 95% CI = (0.01, 0.81)) for each G allele at *COMT* rs6269, and it also increased by 0.55 (*p* = 0.033, 95% CI = (0.05, 1.06)) for each G allele at *TNFSF15* rs4263839.

### 3.6. Associations between Psychological Symptoms and SNP Genotypes

**Figure 3:**
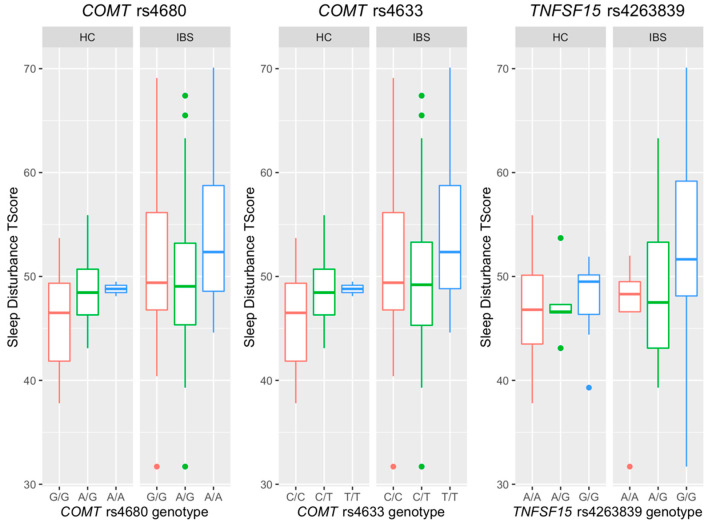
Boxplots for sleep disturbance T-Score in the genotypes of *COMT* rs4680, *COMT* rs4633, and *TNFSF* rs4263839.

Three SNPs were significantly associated with sleep disturbance (*COMT* rs4680, *COMT* rs4633, and *TNFSF15* rs4263839). Figure 3 presents the sleep disturbance scores across different genotypes in these three aforementioned SNPs. Participants who reported higher sleep disturbance scores carried A/A genotype at *COMT* rs4680, T/T genotype at *COMT* rs4633, and G/G genotype at *TNFSF15* rs4263839, respectively. It is worth noting that the plots for *COMT* rs4680 and *COMT* rs4633 are very similar, since these two SNPs are highly correlated due to their close location from 5′ to 3′ in the *COMT* gene [50,51]. The linear regression models for sleep disturbance are shown in Table 3. Sleep disturbance score increased by 1.96 (*p* = 0.051, 95% CI = (−0.01, 3.93)) for each A allele at *COMT* rs4680, by 2.03 (*p* = 0.04, 95% CI = (0.05, 4.01)) for each T allele at *COMT* rs4633, and by 3.28 (*p* = 0.004, 95% CI = (1.08, 5.47)) for each G allele at *TNFSF15* rs4263839.

**Figure 4:**
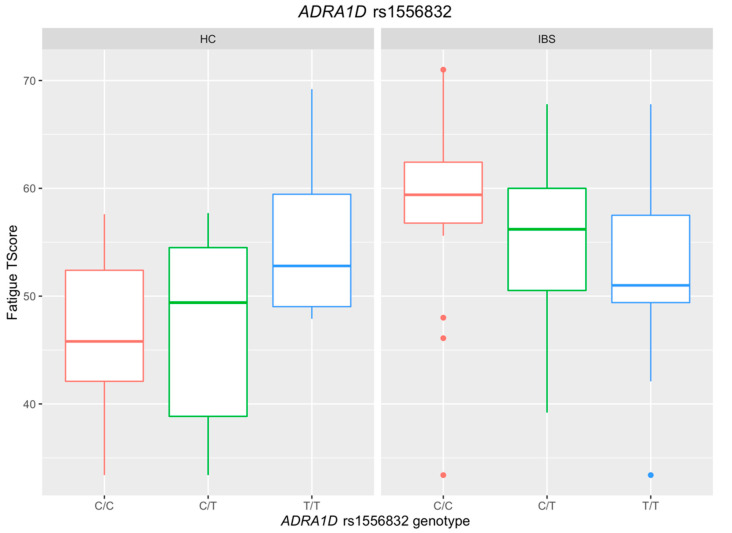
Association between *ADRA1D* rs1556832 and Fatigue in the IBS and the HC groups.

*ADRA1D* rs1556832 SNP genotype was associated with fatigue, but the major allele (C) was the risk allele in the IBS group, while the minor allele (T) conveyed risk for fatigue in the HC group. Figure 4 shows that the participants in the IBS group carrying more C alleles reported a higher fatigue level, while the trend was the opposite in the HC group. Table 3 presents the significant interaction effect of the number of T alleles of *ADRA1D* rs1556832 by groups (*p* = 0.007) on fatigue. Specifically, for each C allele at *ADRA1D* rs1556832, the fatigue score increased by 3.47 (*p* = 0.016, 95% CI = (0.65, 6.28)) among the IBS patients, whereas the fatigue score decreased by 4.27 (*p* = 0.084, 95% CI = (−9.13, 0.58)) in the HC group.

## 4. Discussion

Although IBS is a globally prevalent health problem, the linkage between individual genetic variations with IBS risk and symptom burden is still largely unknown. After the evaluation of eleven candidate SNPs, our study identified five IBS-associated polymorphisms that were involved in neuro-immune signal pathways: *TNFSF15* rs4263839 (cytokines system), *ADRA1D* rs1556832 (adrenergic system), *COMT* (rs4680, rs6269), *SLC6A4* 5-HTTLPR (serotonin reuptake transporter), and *HTR3A* rs1062613 (serotonin system). These findings point out that these SNPs may act alone or may form a multigenic complex to increase the risk of IBS disease and the severity of symptoms, such as abdominal pain, sleep disturbance, and fatigue, mainly through regulating serotonin and adrenergic systems.

### 4.1. Polymorphisms and IBS Risk

Polymorphisms of *TNFSF15* rs4263839, *SLC6A4* 5-HTTLPR, *HTR3A* rs1062613, and *OXTR* rs2254298 were found with increased IBS risk in our study. The G allele of *TNFSF15* rs4263839 was the IBS risk allele that is consistent with other findings conducted in the U.S. and Sweden studies [17]; however, the study did not specify the population’s racial background [17]. Both l and xl alleles of 5-HTTLPR in the *SLC6A4* gene were found more common in IBS participants, and the s allele was the major allele in the HC group in our study, while the s allele of *SLC6A4* 5-HTTLPR and l allele were considered the risk allele of IBS in two other studies, respectively [52,53]. We also found an association between the SNPs of *HTR3A* and IBS risk, consistent with a study in Chinese women [54], but not in the American population [55]. We found that the G allele of *OXTR* rs2254298 was significantly associated with increased IBS risk in the current study.

### 4.2. Polymorphisms and IBS Related Pain

In our study, polymorphisms of *TNFSF15* rs4263839, *COMT* rs6269, and 5-HTTLPR in *SLC6A4* were associated with BPI average pain severity. IBS participants carrying risk (G) allele of *TNFSF15* rs4263839 reported higher BPI average pain than IBS participants with protective allele (A), pointing to the potential for a link between increased TNFSF15 protein expression with IBS risk [17]. Carriers of the risk allele (G) at *COMT* rs6269 had greater pain than the protective allele (A) carriers in IBS participants of our study, consistent with the previous study in patients with sickle cell disease [56]. There was no association between *COMT* rs4680 genotype and BPI average pain in the present study, which was inconsistent with the previous studies of 18 to 44 year-old subjects with thermal pain and pain in geriatric patients with Parkinson’s disease [57,58].

IBS participants of our study who carried the xl allele of *SLC6A4* 5-HTTLPR reported significantly higher pain levels than IBS participants who carried the homozygous s or l allele. Another study found that IBS participants with at least one s allele of *SLC6A4* 5-HTTLPR had a lower thermal pain threshold [59]. Although the mechanism of the association between the l allele and pain burden in IBS patients [60] remains incompletely understood, the s allele has been shown to decrease the transcriptional activity of 5-HTTLPR and hinder the 5-hydroxytryptamine (HT) reuptake process and increase pain sensitivity [59].

### 4.3. Polymorphisms and Sleep Disturbance

In the current study, people with one or more G allele of *TNFSF15* rs4263839 showed an increased chance of developing sleep disturbance, consistent with other findings that blockage TNFSF in patients with high inflammation improve their sleep quality [61]. *TNFSF15* rs4263839 is an intronic variant that may not directly impact the TNFSF15 protein structure or function but can impact its expression.

We also showed that *COMT* rs4680 genotype (Val158Met) was associated with sleep disturbance. This finding is consistent with the result that the Met allele decreases COMT enzymatic activity, resulting in a concomitant increase in prefrontal-dopamine levels, which could cause sleep deprivation [62]. The present study also found a significant association between the *COMT* rs4633 genotype and sleep disturbance, which has not been reported by others. These two SNPs may affect *COMT* mRNA encoding and protein expression individually or in combination. The enzymatic changes of *COMT* may impact deactivating catechol substrates (epinephrine, norepinephrine, and dopamine) and therefore mediate behavioral functions such as sleep disturbance [50].

### 4.4. Polymorphisms and Fatigue

To the best of our knowledge, the current study is the first to report the association between the polymorphic variant of *ADRA1D* and fatigue in IBS participants. Fatigue-related risk alleles of *ADRA1D* rs1556832 were found to be different between the healthy control (T allele) and IBS (C allele) participants. These findings are consistent with a prior report that *ADRA1D* rs1556832 major allele homozygotes (C/C) in IBS have increased GI symptom severity and brain morphological changes [24]. Therefore, the risk allele could be used to predict IBS symptom severity and neurological or psychological disorders. The mechanisms underlying the association between minor allele (T allele) and increased fatigue in the HC group is still unknown, and the small sample size makes additional interpretation challenging; therefore, further investigation is warranted.

Although this study helps us understand the impact of genetic factors on IBS risks and symptoms, it is important to realize that the genetic information alone may not fully explain the etiology of IBS. Environmental factors such as pollution, diet, exercise, and other health conditions also contribute to IBS development and severity [63]. Further studies need to investigate the multifactorial pathogenesis of IBS.

### 4.5. Limitations

The majority of our study subjects were non-Hispanic white young adults, which may affect the generalizability of the results. The small sample size of both the HC and IBS groups may lead to biased results. A conservative multiplicity adjustment to control the family-wise Type I error rate was not applied, as is the nature of an exploratory study. Our study did not analyze gene or protein expression, which may hinder the genetic linkage between SNPs and molecular mechanisms’ variation. Further studies with a large sample size are needed to explore the relationship between the variation of SNPs, gene, protein, IBS risk, and severity of symptoms.

## 5. Conclusions

The present study demonstrates genetic variations of *HTR3A* rs1062613, *SLC6A4* 5-HTTLPR, *COMT* (rs4680, rs6269), and *ADRA1D* rs1556832, and *TNFSF15* rs4263839 may contribute to a multigenic risk profile for IBS diagnosis and symptom burden. These results must be replicated in a larger population to predict IBS susceptibility and disease severity in order to potentially assist in clinical intervention and treatment decision making. This study points to the opportunities for applying genetic analysis to the development of individualized treatment plans for IBS pain.

## Figures and Tables

**Table 1:** Demographic characters in irritable bowel syndrome (IBS) and healthy control (HC) groups.

Demographic		*n*	HC (*n* = 21)	IBS (*n* = 80)	*p*-Value
**Gender**					
Female		72	11 (52.38%)	61 (76.25%)	0.060
Male		29	10 (47.62%)	19 (23.75%)	
**Race**					
White		73	11 (52.38%)	62 (77.50%)	0.070
Asian		16	6 (28.57%)	10 (12.50%)	
Black or African American		12	4 (19.05%)	8 (10.00%)	
**Ethnicity**					
Not Hispanic or Latino		84	16 (76.19%)	68 (85.00%)	0.360
Hispanic or Latino		11	4 (19.05%)	7 (8.75%)	
Not reported		6	1 (4.76%)	5 (6.25%)	
**Education**					
High school or lower		8	2 (9.52%)	6 (7.50%)	0.151
Some college, no degree Associate degree: academic program		63 3	16 (76.19%) 1 (4.76%)	47 (58.75%) 1 (1.25%)	
Bachelor degree		16	2 (9.52%)	14 (17.50%)	
Master degree		12	0 (0.00%)	12 (15.00%)	
**Caregiver Primary Type**					
Parent or legal guardian		53	14 (66.67%)	39 (48.75%)	0.117
Self		46	6 (28.57%)	40 (50.00%)	
Other		2	1 (4.76%)	1 (1.25%)	
**Employment Status**					
Student		75	18 (85.71%)	57 (71.250%)	0.269
Working now		22	2 (9.52%)	20 (25.00%)	
**Looking for work, Unemployed**		4	1 (4.76%)	3 (3.750%)	
Marital Status					
Never married		98	21 (100.00%)	77 (96.25%)	1
Married		3	0 (0.00%)	3 (3.75%)	
	**Mean (SD)**		**Median (Range)**		
	**HC**	**IBS**	**HC**	**IBS**	***p*-Value**
**Age**	20.14 (1.39)	20.39 (2.57)	20 (18–28)	21 (18–23)	0.0717

**Table 2:** Pain and psychological symptom measurements.

	Mean (SD)		Median (Range)		*p*-Value
	HC	IBS	HC	IBS	
Average pain	1.333 (2.869)	10.537 (5.684)	0 (0–12)	9.500 (2–29)	1.6 × 10^−10^
Pain interference	0.231 (0.395)	2.177 (1.860)	0 (0–1.1)	1.571 (0–8.3)	5.1 × 10^−9^
Anxiety	54.548 (5.999)	59.975 (8.786)	55.800 (39.1–65.0)	61.150 (39.1–82.4)	0.005
Cognition	35.776 (4.788)	35.959 (7.155)	35.300 (28.3–43.4)	36.050 (24.8–49.2)	0.844
Depression	48.481 (6.119)	51.251 (8.914)	50.700 (38.4–58.4)	52.150 (38.4–66.9)	0.160
Fatigue	48.733 (9.212)	55.246 (8.169)	49.400 (33.4–69.2)	56.800 (33.4–71.0)	0.003
Sleep disturbance	47.676 (4.334)	51.153 (7.825)	48.200 (37.8–55.9)	49.800 (31.7–70.1)	0.051

**Table 3:** Results of linear regression models for pain and psychological symptom measurements vs. SNPs.

	Coefficient	95% CI	*p*-Value
BPI Average Pain			
*SLC6A4* 5-HTTLPR xl (reference = s + l)	1.24	(0.27, 2.22)	0.013
*COMT* rs6269 G	0.41	(0.01, 0.81)	0.045
*TNFSF15* rs4263839 G	0.55	(0.05, 1.06)	0.033
Sleep Disturbance T-Score			
*COMT* rs4680 A	1.96	(−0.01, 3.93)	0.051
*COMT* rs4633 T	2.03	(0.05, 4.01)	0.044
*TNFSF15* rs4263839 G	3.28	(1.08, 5.47)	0.004
Fatigue T-Score			
*ADRA1D* rs1556832 C * Group IBS	7.74	(2.18, 13.30)	0.007
*ADRA1D* rs1556832 C in IBS	3.47	(0.65, 6.28)	0.016
*ADRA1D* rs1556832 C in HC	−4.27	(−9.13, 0.58)	0.084

The models adjusted the effects of gender, race, ethnicity, and the type of caregiver; *ADRA1D* rs1556832 C * Group IBS represent the interaction term between the two variables.

## Supplementary Materials

The following supporting information can be downloaded at: https://www.mdpi.com/article/10.3390/jpm12020142/s1. Table S1. Frequency and proportion of genotypes of the 11 polymorphisms in IBS and HC groups (*N* = 101).
