# Supplementary material for: The Associations of Single Nucleotide Polymorphisms with Risk and Symptoms of Irritable Bowel Syndrome
Source: J Pers Med. 2022 Jan 21;12(2):142. doi: 10.3390/jpm12020142 (PMC8878682; doi:10.3390/jpm12020142)
Supplement: Supplementary file 1 [file jpm-12-00142-s001.zip › jpm-1541026-supplementary.pdf]

Supplemental Table S1. Frequency and proportion of genotypes of the 11 polymorphisms in IBS and HC groups (N = 101).

Polymorphisms	Genotype	IBS n(%)	HC n(%)	Fisher's exact test p-value	Chi-square test p-value of HWE	
					IBS	HC
SLC6A4 5-HTTLPR	S	18 (22.5%)	20 (95.24%)	< .001	NA [b]	NA [b]
	L	53 (66.25%)	1 (4.76%)			
	xl	8 (10%)	0 (0%)			
	No call	1 (1.25%)	0 (0%)			
ADRAID rs1556832	C/C	18 (22.5%)	9 (42.86%)	.129	0.369	0.437
	C/T	44 (55%)	7 (33.33%)			
	T/T	17 (21.25%)	4 (19.05%)			
	No call	1 (1.25%)	1 (4.76%)			
COMT rs4680	A/A	16 (20%)	2 (9.52%)	.492 [a]	0.089	0.531
	A/G	30 (37.5%)	12 (57.14%)			
	G/G	34 (42.5%)	7 (33.33%)			
COMT rs4818	C/C	29 (36.25%)	12 (57.14%)	.492 [a]	0.170	0.823
	C/G	32 (40%)	7 (33.33%)			
	G/G	18 (22.5%)	2 (9.52%)			
	No call	1 (1.25%)	0 (0%)			
COMT rs6269	A/A	27 (33.75%)	7 (33.33%)	.553 [a]	0.334	0.838
	A/G	34 (42.5%)	10 (47.62%)			
	G/G	18 (22.5%)	3 (14.29%)			
	No call	1 (1.25%)	1 (4.76%)			
COMT rs4633	T/T	16 (20%)	2 (9.52%)	.492 [a]	0.068	0.531
	C/T	29 (36.25%)	12 (57.14%)			
	C/C	34 (42.5%)	7 (33.33%)			
	No call	1 (1.25%)	0 (0%)			
HTR3A rs1062613	C/C	50 (62.5%)	13 (61.9%)	.014	< 0.001	< 0.001
	C/T	16 (20%)	0 (0%)			
	T/T	11 (13.75%)	8 (38.1%)			
	No call	3 (3.75%)	0 (0%)			
OPRM1 rs1799971	G/G	5 (6.25%)	3 (14.29%)	.637	0.047	0.091
	A/G	16 (20%)	4 (19.05%)			
	A/A	58 (72.5%)	14 (66.67%)			
	No call	1 (1.25%)	0 (0%)			
OXTR rs53576	G/G	36 (45%)	5 (23.81%)	.158 [a]	0.595	0.722
	A/G	36 (45%)	12 (57.14%)			
	A/A	6 (7.5%)	4 (19.05%)			
	No call	2 (2.5%)	0 (0%)			
OXTR rs2254298	G/G	60 (75%)	11 (52.38%)	.076 [a]	0.904	0.859
	A/G	19 (23.75%)	8 (38.1%)			
	A/A	1 (1.25%)	2 (9.52%)			
TNFSF15 rs4263839	G/G	50 (62.5%)	11 (52.38%)	.074	0.616	0.126
	A/G	25 (31.25%)	5 (23.81%)			
	A/A	5 (6.25%)	4 (19.05%)			
	No call	0 (0%)	1 (4.76%)			

Note: [a] p-values adjusted by Holm-Bonferroni correction for the SNPs on the same gene; [b] Not Applicable.
